# Enzymatic Degradation Behavior and Molecular Weight Regulation of Dextran: Empirical Modeling and Multi-Scale Structural Characterization

**DOI:** 10.3390/cimb48070749

**Published:** 2026-07-22

**Authors:** Mei Li, Piaoran Fan, Yirui Zhang, Ranran Li, Lemin Chen, Donghui Zhang, Lei Zhong

**Affiliations:** Guangxi Key Laboratory of Polysaccharide Materials and Modification, School of Chemistry and Chemical Engineering, Guangxi Minzu University, Nanning 530006, China; meili@gxmzu.edu.cn (M.L.); 15516442022@163.com (P.F.); 2023200216@snnu.edu.cn (Y.Z.); lr051200@163.com (R.L.); cleminn@163.com (L.C.); 15232157763@163.com (D.Z.)

**Keywords:** enzymatic degradation, dextran, low-molecular-weight dextran, process optimization, quality control

## Abstract

To meet the demand for controlled production of low-molecular-weight (*M*_w_ < 10 kDa) dextran with potential pharmaceutical applications, this study developed an efficient enzymatic preparation process using PC-Edex, a dextranase derived from *Penicillium cyclopium* CICC-4022. The effects of enzyme concentration, substrate concentration, temperature, and pH on the degradation of high-molecular-weight dextran were systematically investigated, and the optimal process conditions were established. A staged empirical control strategy based on the Malhotra model was developed to investigate and predict the behavior of dextran molecular weight changes during enzymatic hydrolysis. Under the optimized conditions, dextran with an *M*_w_ below 10 kDa was produced within 60 min, with the mass fraction of fragments smaller than 10 kDa reaching 94.56 ± 0.32% and the degradation rate exceeding 98.96 ± 0.15%. The resulting product exhibited a narrow molecular weight distribution (*M*_w_/*M*_n_ = 1.528 ± 0.03) and adopted a compact random-coil conformation in aqueous solution. Multi-scale characterization results indicated that enzymatic degradation altered only the molecular weight of dextran, while the backbone structure, amorphous nature, and thermal stability were preserved. These findings present a robust and reproducible laboratory-scale process, which provides a reference for the industrial production of low-molecular-weight dextran for pharmaceutical purposes.

## 1. Introduction

Dextran is a polysaccharide composed primarily of α-(1 → 6) glycosidic-linked D-glucose units, exhibiting excellent biocompatibility and biodegradability [1,2]. High-molecular-weight dextran can be chemically or biologically modified to yield novel materials and functional chemicals with enhanced properties, such as dextran microspheres, stimuli-responsive dextran hydrogels, targeted drugs, and drug carriers. Collectively, these derivatives demonstrate broad application potential across industrial and medical fields [3,4]. High-molecular-weight dextran (*M*_w_ > 1000 kDa) is frequently used as a chromatography column filler [5]; dextran within the *M*_w_ range of 100–1000 kDa serves as an emulsifier, stabilizer, and thickener in food products [6]; dextran within the *M*_w_ range of 10–100 kDa (including 20–70 kDa) functions as a plasma substitute for clinical volume expansion [7]; and low-molecular-weight dextran (*M*_w_ < 10 kDa), including variants of *M*_w_ 6–8 kDa fraction, is the core raw material for iron dextran preparations used to treat iron deficiency anemia [8]. As a biopolymer produced through microbial fermentation by bacterial species such as *Leuconostoc mesenteroides*, dextran represents a typical high-molecular-weight carbohydrate [9]. However, fermentation-derived dextran has an extremely high initial molecular weight and broad molecular weight distribution (*M*_w_/*M*_n_), which cannot be directly used in pharmaceutical applications and may induce adverse clinical effects such as renal toxicity and allergic reactions [10].

For low-*M*_w_ dextran intended for pharmaceutical use, clinical safety is critically dependent on precise control of molecular weight and its distribution. Key quality control indicators include weight-average molecular weight (*M_w_*), number-average molecular weight (*M*_n_), polydispersity index (*M*_w_/*M*_n_), solution conformation, and structural integrity [11]. A well-established multi-scale analytical system combining gel permeation chromatography coupled with multi-angle light scattering, refractive index and viscometric detection (GPC-MALS-RI-Vis), atomic force microscopy (AFM), scanning electron microscopy (SEM), Fourier transform infrared spectroscopy (FT-IR) and nuclear magnetic resonance (NMR) is required to ensure that the product meets pharmacopoeia standards. However, most existing studies on dextran degradation have focused primarily on molecular weight reduction, with limited systematic research on the establishment of industrially applicable process parameters and complete quality control systems.

The degradation of high-molecular-weight dextran into low-molecular-weight dextran can be achieved through enzymatic, acid-based, or ultrasonic methods. Acid-based degradation, the current mainstream industrial process, has severe disadvantages, including harsh reaction conditions, serious chlorine residue contamination, and the discharge of large amounts of acidic wastewater [12]. Ultrasonication fails to fully degrade high-*M*_w_ dextran (>100 kDa) and is associated with high energy consumption, making it unsuitable for large-scale production [13]. In contrast, enzymatic degradation demonstrates significant application value due to its mild reaction conditions, high specificity, and environmental friendliness [14]. Endo-dextranase specifically cleaves α-(1 → 6) glycosidic bonds in dextran, producing uniform-*M*_w_ functional dextran, making it the most promising alternative for controlled low-*M*_w_ dextran production [15].

Previously, our research group identified the fungal strain *Penicillium cyclopium* CICC-4022, which exhibits high dextranase (PC-Edex) production capacity and shows good potential for generating low-*M*_w_ dextran with a narrow molecular weight distribution [16,17]. Current research on low-*M*_w_ dextran production predominantly focuses on (1) dextranase purification, (2) enzymatic characterization, (3) degradation product identification, and (4) preliminary enzymatic regulation of dextran molecular weight [18]. Recent studies have established that enzymatic dextran degradation follows certain empirical degradation patterns, but most of these studies have used dextran substrates with initial *M*_w_ substantially lower than those of fermentation-derived dextran [19,20]. Consequently, there remains a lack of systematic process optimization and molecular weight control methods for producing controlled low-*M*_w_ dextran with potential clinical value from high-*M*_w_ fermentation-derived dextran.

The present study aimed to develop an efficient, reproducible enzymatic process with industrial application potential, for preparing controlled low-*M*_w_ dextran (<10 kDa) with potential clinical application value using PC-Edex. High-*M_w_* fermentation-derived dextran served as the model substrate for systematically optimizing key process parameters, including enzyme concentration, substrate concentration, temperature and pH. A staged empirical regulation method for molecular weight control was established, and the quality of the prepared low-*M*_w_ dextran was comprehensively characterized using a multi-scale analytical system. These findings present a promising laboratory-scale enzymatic strategy, provide a theoretical basis for controlled low-*M*_w_ dextran production, and offer a reference for future industrial scale-up.

## 2. Materials and Methods

### 2.1. Materials

Dextran-T70 and Dextran-T100 (weight-average molecular weight, *M*_w_ = 69.23 kDa and 138.55 kDa, respectively) were purchased from Solarbio (Beijing, China). High-molecular-weight dextran (Dextran-H; *M*_w_ = 4167.16 kDa) was obtained from BioBioMei (Hefei, China). Strains of *P. cyclopium* CICC-4022 and *L. mesenteroides* CICC 21725 were acquired from the China Center for Industrial Culture Collection (Beijing, China). PC-Edex was produced and purified from *P. cyclopium* CICC-4022, while Dextran-F (*M*_w_ = 5847.61 kDa) was biosynthesized and purified from *L. mesenteroides* CICC 21725 following procedures detailed in Supplementary Notes S1 and S2 [21]. All other chemicals were analytical grade from Sinopharm Chemical Reagent Co., Ltd. (Shanghai, China). Reported molecular weights represent weight-average (*M*_w_) values.

### 2.2. Matrix-Assisted Laser Desorption Ionization Tandem Time-of-Flight Mass Spectrometry (MALDI-TOF)

An aliquot of the PC-Edex solution (10 mg/mL) was diluted with trifluoroacetic acid. The matrix used was a saturated solution of sinapinic acid prepared in 50% (*v*/*v*) acetonitrile/water. Following molecular weight (*M*_w_) calibration using Protein Calibration Standards II (P/N 8207234), analysis was performed on a Bruker autoflex maX mass spectrometer (Bruker Corporation, Billerica, MA, USA) operated in positive linear mode.

### 2.3. Enzymatic Hydrolysis of Dextran for Controlled M_w_ Products

#### 2.3.1. Chromatographic Conditions

The chromatographic conditions were as follows: columns, Ultrahydrogel^TM^ 2000, Ultrahydrogel^TM^ 250, and Ultrahydrogel^TM^ DP120A (each 7.8 mm × 300 mm; Shimadzu, Kyoto, Japan); mobile phase, 0.03% NaN_3_ and 0.1 M NaNO_3_; flow rate, 0.5 mL/min; injection volume, 0.2 mL; detectors, Waters 2414 refractive index detector (Waters Corporation, Milford, MA, USA), Viscostar^TM^ viscometer, and multi-angle light scattering (MALS) detector (Wyatt Technology Corporation, Santa Barbara, CA, USA); column and detector temperature, 35 °C; refractive index increment (dn/dc), 0.138 mL/g.

#### 2.3.2. Dextran Hydrolysis with PC-Edex

Single-factor screening experiments were first performed to investigate the effects of PC-Edex concentration (2, 4, 8, 12 U/mL), Dextran-H substrate concentration (10, 30, 50, 70 mg/mL), the pH of the 20 mM acetic acid buffer solution (4.0, 5.0, 6.0, 7.0), and temperature (40, 45, 50, 55 °C) on the molecular weight (*M*_w_) of Dextran-H hydrolysis products. These screening assays were conducted as single-run measurements (*n* = 1) to preliminarily narrow down the optimal parameter range. The optimized parameters were subsequently used for triplicate validation experiments (*n* = 3) and for assessing the degradation of Dextran-T70, Dextran-T100, Dextran-H, and Dextran-F by PC-Edex. Briefly, PC-Edex and dextran were combined at a 1:1 (*v*/*v*) ratio. Aliquots were withdrawn at specific time intervals, and reactions were terminated by immersing samples in a boiling water bath for 5 min. This boiling treatment ensures complete and irreversible inactivation of PC-Edex based on its reported thermal stability properties. The mobile phase was diluted tenfold and filtered through a 0.22-μm aqueous membrane. The weight-average molecular weight (*M*_w_) and dispersity index (*M*_w_/M_n_) of dextran were determined using GPC-MALS-RI-Vis (Waters Corporation, USA; Wyatt Technology Corporation, USA) under the conditions specified in Section 2.4.1.

#### 2.3.3. Malhotra Model

The variation of the *M*_w_ of dextran with time (t) was expressed by the following equation:

(1) $$\frac{d\left[M_{w}\right]}{dt}=-k{\left[M_{w}\right]}^{n}$$

When n = 1, the polymer followed a first-order hydrolysis, which then required the following formulas:

(2) $$\int_{\left[M_{w,0}\right]}^{\left[M_{w,t}\right]}\frac{1}{\left[M_{w}\right]}d\left[M_{w}\right]=-k\int_{0}^{t}dt$$

(3) $$\ln M_{w,t}=\ln M_{w,0}-kt$$

When n = 2, the polymer followed a second-order hydrolysis, which then required the following formulas:

(4) $$\int_{\left[M_{w,0}\right]}^{\left[M_{w,t}\right]}\frac{1}{{\left[M_{w}\right]}^{2}}d\left[M_{w}\right]=-k\int_{0}^{t}dt$$

(5) $$\frac{1}{M_{w,t}}=\frac{1}{M_{w,0}}+kt\;a$$

where *M*_w,0_ indicates the initial *M*_w_ of the polymer, and *M*_w,t_ indicates the *M*_w_ of the polymer at a certain treatment time.

Moreover, the evolution of the molecular weight (*M*_w_) of dextran degraded by PC-Edex was modeled using the Malhotra equation [22] (Equation (5)). Degradation kinetics were further evaluated by calculating the coefficient of determination (R^2^). In this study, R^2^ > 0.98 was defined as the threshold for high goodness of fit, which indicates strong consistency between the experimental data and the model.

#### 2.3.4. Hydrolysis Rate of Dextran

The average enzymatic hydrolysis rate of dextran per unit time was calculated using Equation (6) as shown below:

(6) $$Degradation\;rate\left(\%\right)=\frac{M_{w,0}-M_{w,t}}{M_{w,0}}\times 100\%$$

where *M*_w,0_ indicates the initial *M*_w_ of the polymer, and *M*_w,t_ indicates the *M*_w_ of the polymer at a certain point of hydrolysis treatment [23].

#### 2.3.5. Determination of Mass Fraction of Dextran at Different *M*_w_

The mass fractions of dextran across distinct molecular weight ranges were determined using ASTRA 7.1.3 software (Wyatt Technology LLC., Santa Barbara, CA, USA) within the GPC-MALS-RI-Vis system. The molecular weight distribution profiles provided the dispersity (*M*_w_/*M*_n_) for each sample and enabled quantification of molar mass fractions as percentages of the total distribution.

#### 2.3.6. Determination of PC-Edex Enzymatic Activity

The enzymatic activity of PC-Edex was determined via the 3,5-dinitrosalicylic acid (DNS) method using Dextran-T70 as the substrate. All solutions were prepared with 20 mM acetate buffer (pH 5.0). Briefly, 1 mL of PC-Edex solution was mixed with 1 mL of 30 mg/mL Dextran-T70 substrate solution, and the mixture was incubated in a 55 °C water bath for 10 min. The reaction was terminated by immersion in a boiling water bath for 10 min. Then, 1 mL of the reaction mixture was mixed with 1 mL of DNS reagent, followed by color development in a boiling water bath for 10 min. The absorbance was measured at 540 nm. A control group was set up with heat-inactivated PC-Edex under identical conditions.

A unit of PC-Edex activity (1U) was determined based on the amount of PC-Edex to degrade dextran T70 and produce 1 mg of reducing sugar within 1 h [24,25]. PC-Edex activity was calculated based on Equation (7) as follows: [26]

(7) $$\begin{array}{c} PC-\mathrm{Edex}\;\mathrm{activity}\left(\frac{U}{mL}\right)=\\ \frac{Amount\;of\;reducing\;sugar\left(mg\right)\times Dilution\;factor\;of\;PC-Edex\;solution}{Volume\;of\;PC-Edex\left(mL\right)\times Time\left(h\right)} \end{array}$$

Protein concentration (mg/mL) was determined utilizing the Bradford method and crystallized bovine serum albumin as the protein standard [27].

### 2.4. Analysis of the Molecular Structure of Dextran After Enzymatic Hydrolysis

#### 2.4.1. Analysis of the Conformational Structure of Chains in Solution

GPC-MALS-RI-Vis was used as the main technique to determine the chain of dextran at different *M*_w_ in solution based on the hydrodynamic radius (Rh) versus *M*_w_ and the characteristic viscosity [η] versus *M*_w_ curves.

#### 2.4.2. Atomic Force Microscopy (AFM)

Briefly, 5 μL of dextran solution (10 ng/mL) of varying *M*_w_ was placed and left to dry on MICA sheets. A MultiMode8 atomic force microscope (Bruker Corporation, Billerica, MA, USA) was used to scan the morphology of dextran of varying *M*_w_.

#### 2.4.3. Scanning Electron Microscopy (SEM)

A small amount of dextran powder of varying *M*_w_ was placed upon a conductive tape, and a gold-plating machine was used for platinum spraying. After cooling, samples were observed in a ZEISS Gemini 300 scanning electron microscopy (Carl Zeiss Microscopy GmbH, Jena, Germany).

#### 2.4.4. Fourier Transform Infrared Spectroscopy (FT-IR)

Nicolet^TM^ iS^TM^ 10 FT-IR (Thermo Fisher Scientific, Waltham, MA, USA) was used to obtain FT-IR spectra of dextran samples with a resolution of 8 cm^−1^ within the range of 400–4000 cm^−1^. Precipitates of dextran of different *M*_w_ were mixed with KBr at a ratio of 1:100 (*w*/*w*), and then pressed into a metal mold to obtain testing disks.

#### 2.4.5. Nuclear Magnetic Resonance (NMR)

Powder of dextran of different *M*_w_ was dissolved in 99.96% D_2_O, and ^1^H and ^13^C NMR analysis was conducted to characterize the structure of dextran in a Bruker 400 NMR spectroscope (Bruker Scientific Instruments, Billerica, MA, USA).

#### 2.4.6. Isothermal Titration Calorimetry (ITC) Assay

The interaction behavior of PC-Edex with Dextran-T70 was qualitatively investigated by ITC (ITC 200, Microcal, Inc., Northampton, MA, USA). The stirring speed was set at 150 rpm. All solutions were prepared in acetate buffer at pH 5.0 (20 mmol L^−1^).

Interactions were assessed by two different methods: titration of PC-Edex with Dextran-T70 at different temperatures (40, 45, 50, 55 °C) and titration of PC-Edex with different molecular weight dextran substrates (Dextran-T20, Dextran-T70, Dextran-T500, Dextran-T2000) at 55 °C. To assess the molecular interaction of the enzyme with the substrate, 50 µL of dextran (0.429 mmol L^−1^) was injected in the syringe and 350 µL of PC-Edex enzyme solution (0.5 µmol L^−1^) was injected in the sample cells.

For all ITC assays, automatic titration of dextran was started after the calorimeter reached the experimental temperature and the baseline was stabilized. The volume of each drop was 1 µL for a total of 20 drops with an injection interval of 60 s. All results obtained were discounted against a blank assay.

The single-injection method [28] was used to fit the experimental data solely to obtain Michaelis–Menten for investigating the catalytic behavior of PC-Edex and guiding process optimization, rather than for quantitative thermodynamic analysis of enzyme–substrate binding or for inferring molecular mechanisms. The instantaneous catalytic reaction rate (*v*, mol/(s·L)) can be expressed as Equation (8). Under identical experimental conditions, v maintains a fixed proportional relationship with the ITC-measured thermal power (*P*, J/s), so the reaction rate can be visualized directly from the heat flow curve. The variation of substrate concentration ([*S*], mol/L) in the reaction cell with volume *V* (L) is presented in Equation (9). The Michaelis–Menten equation for calculating the initial reaction rate (*v*_1_, mol/(s·L)) is shown in Equation (10), where *k*_cat_ (s^−1^) is the catalytic constant of PC-Edex, [*E*] (mol/L) is the enzyme concentration in the system, and *K*_m_ (mol/L) is the Michaelis constant of the enzyme.

(8) $$v=-\frac{d\left[S\right]}{dt}=\frac{P}{∆H_{hydr}V}$$

(9) $$\left[S\right]=\left(S_{0}\right)-\frac{\int_{0}^{t}Pdt}{∆H_{hydr}V}$$

(10) $$v_{1}=\frac{k_{cat}\left[E\right]\left[S\right]}{K_{m}+\left[S\right]}$$

### 2.5. Statistical Analysis

The experimental design in this study consists of two independent phases: parameter screening and condition validation. Single-factor optimization experiments (enzyme concentration, substrate concentration, temperature, and pH) were conducted as single-run screening assays (*n* = 1) to preliminarily identify the optimal parameter range. All validation experiments under the finalized optimal reaction conditions were performed in triplicate (*n* = 3). Data from validation experiments are presented as mean ± standard deviation (SD). Statistical significance was evaluated using one-way ANOVA followed by Tukey’s multiple comparison test. A value of *p* < 0.05 was considered statistically significant.

## 3. Results

### 3.1. MALDI-TOF Analysis

**Figure 1:**
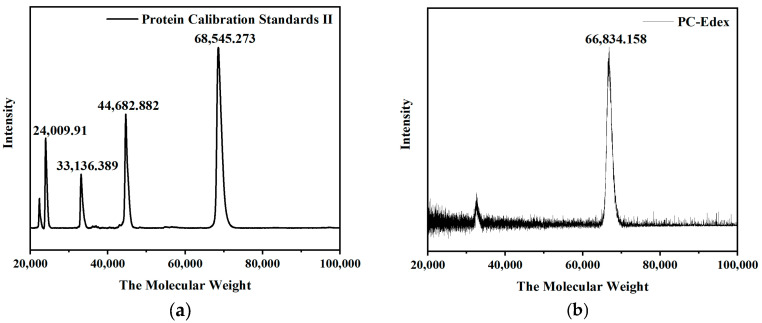
Mass spectra of (**a**) Protein Calibration Standards II and (**b**) PC-Edex, an extracellular endo-dextranase purified from the strain *P. cyclopium* CICC-4022.

Previous studies indicate that fungal dextranase exhibits a molecular weight (*M*_w_) range of 40–80 kDa [29,30]. Purification data for PC-Edex are presented in Table S1. As shown in Figure S1, SDS-PAGE analysis estimated the *M*_w_ of PC-Edex at 77 kDa. The discrepancy between SDS-PAGE and MALDI-TOF results may be attributed to glycosylation modifications of PC-Edex. As a fungal dextranase, PC-Edex likely carries carbohydrate modifications that increase its hydrodynamic radius in the SDS-PAGE system and reduce electrophoretic mobility, leading to overestimated molecular weight relative to non-glycosylated calibration standards. Given that SDS-PAGE may introduce errors in protein *M*_w_ determination [31], MALDI-TOF mass spectrometry was employed for verification, yielding a refined *M*_w_ of 66.83 kDa (Figure 1).

### 3.2. Hydrolysis of Dextran by PC-Edex

#### 3.2.1. Effect of PC-Edex Activity on the *M*_w_ of Dextran-H

As described earlier, dextran at different *M*_w_ has different applications. In previous studies, it was shown that enzymatic hydrolysis of dextran is the best method to obtain low-*M*_w_ dextran (*M*_w_ < 10 kDa) [19,23]. Table S2 shows the variation of *M*_w_ and *M*_w_/*M*_n_ of the PC-Edex-degraded substrate Dextran-H over time at different PC-Edex activities. Changes in the *M*_w_ of Dextran-H accelerated as the relative PC-Edex activity increased. When PC-Edex was added at 8 U/mL for 30–60 min, the *M*_w_ of Dextran-H remained relatively stable within the low *M*_w_ range; when PC-Edex was added at 2–4 U/mL, slow *M*_w_ change was observed; when PC-Edex was added at 12 U/mL, fast *M*_w_ change occurred. PC-Edex could, with reaction time, degrade high-*M*_w_ Dextran-H into low-*M*_w_ dextran, indicating that PC-Edex had a high catalytic effect. The enzymatic degradation of Dextran-H is usually terminated by high temperature; thus, an excessively high PC-Edex activity is undesirable, since it can result in difficulties in controlling the *M*_w_ change of Dextran-H.

It is known that the *M*_w_ of a polysaccharide is dynamic within a range of *M*_w_/*M*_n_. *M*_w_/*M*_n_ reflects the homogeneity of a polymer’s molecular weight distribution; a value closer to 1 indicates higher homogeneity [32]. As shown in Table S2, elevated *M*_w_/*M*_n_ values were observed during the initial stage of Dextran-H degradation, demonstrating heterogeneous molecular weight distribution at this phase. With prolonged degradation time, the *M*_w_/*M*_n_ values decreased progressively, indicating gradual homogenization of the molecular weight distribution at later stages. Collectively, enzymatic degradation using PC-Edex at a concentration of 8 U/mL was selected as the optimal condition from the screening assays for producing target low-*M*_w_ dextran (defined herein as <10 kDa) with homogeneous molecular weight distribution, as it balances hydrolysis efficiency and controlled degradation. Higher enzyme concentrations (e.g., 12 U/mL) resulted in over-degradation, broader molecular weight distribution, and reduced yield of the target < 10 kDa fraction. It should be noted that the data in Table S2 correspond to single-run screening results (*n* = 1); the performance under this optimal condition was further verified in triplicate validation experiments.

From a kinetic perspective, the increase in hydrolysis rate with increasing PC-Edex concentration is consistent with general enzymatic behavior. Within the tested range of 2–12 U/mL, no distinct saturation plateau was observed, suggesting that substrate availability was not rate-limiting under the conditions employed (30 mg/mL dextran). The pronounced excessive hydrolysis at 12 U/mL can be attributed to the high density of catalytic sites, which rapidly cleave internal α-(1 → 6) glycosidic bonds and reduce *M*_w_ below the target range within the standard reaction window. It should be noted that mass transfer effects are likely negligible at the dilute substrate concentrations used here.

#### 3.2.2. Effect of Substrate Concentration on *M*_w_ Change of Dextran-H

Table S3 depicts changes in *M*_w_ and *M*_w_/*M*_n_ over time during the degradation of Dextran-H by PC-Edex at varying substrate concentrations. The concentration of Dextran-H significantly influenced both the enzymatic degradation efficiency and the control of the resulting molecular weight (*M*_w_). Specifically, the rate of *M*_w_ decrease diminished with increasing Dextran-H concentration. This phenomenon primarily arises from the solution behavior of dextran [33], where elevated concentrations increase viscosity, thereby impeding sufficient contact between PC-Edex and Dextran-H. In dilute solutions, dextran chains extend freely, facilitating complete enzymatic reaction and more efficient degradation. As shown in Table S3, at 10 mg/mL within 12–30 min, the *M*_w_ of Dextran-H ranged between 2.83 and 8.23 kDa, corresponding to a rapid *M*_w_ reduction rate but poor molecular weight control upon reaction termination. Conversely, solutions at 50 mg/mL and 70 mg/mL exhibited higher viscosity and poorer mass transfer, limiting the enzyme–substrate interaction. Therefore, a Dextran-H concentration of 30 mg/mL was selected as optimal for achieving enhanced hydrolysis efficiency. In addition to viscosity effects, steric hindrance arising from chain entanglement of high-*M*_w_ dextran at elevated concentrations further restricts access of the endo-dextranase to internal α-(1 → 6) glycosidic bonds.

#### 3.2.3. Effect of Temperature on *M*_w_ Change of Dextran-H

Table S4 depicts the variation of *M*_w_ and *M*_w_/*M*_n_ over time for the degradation of Dextran-H by PC-Edex under the temperature range of 40–55 °C. In this context, the higher the temperature, the faster the rate of decrease in the *M*_w_ of Dextran-H. As demonstrated, temperature strongly influenced the efficiency of enzymatic hydrolysis; at high temperatures, PC-Edex inactivation occurs, whereas at low temperature, PC-Edex activity decreases. Moreover, an appropriate increase in temperature helps to reduce the viscosity of the substrate Dextran-H, thereby promoting the reaction. Thus, selecting the optimum temperature is critical for the enzymatic degradation of Dextran-H. In addition, the thermal stability of PC-Edex at 50 °C was better than that at 55 °C [17]. As summarized in Table S4, since both instantaneous reaction rate and long-term thermal stability of PC-Edex must be taken into consideration for practical degradation processes, 50 °C was selected as the optimal reaction temperature for the practical degradation process.

Isothermal titration calorimetry (ITC) was employed to experimentally observe the heat flow behavior during the interaction of PC-Edex with dextran at different temperatures. As shown in Table S5, the enzyme–substrate binding affinity was highest at 55 °C, as reflected by the strongest heat flow signal during the hydrolysis reaction, which was consistent with the enzyme activity assay results. This result indicates that 55 °C is the temperature optimal for enzyme–substrate binding affinity, at which the favorable contributions from hydrophobic interactions and conformational entropy are maximized. The discrepancy between the binding-optimal temperature (55 °C) and the process-optimal temperature (50 °C) is attributed to the reduced long-term thermal stability of PC-Edex at 55 °C, which would compromise overall degradation efficiency in extended reactions. Consistent with previous molecular docking results [34], the binding is governed by a combination of multiple weak intermolecular interactions, including hydrogen bonds and hydrophobic interactions. Key residues contributing significantly to dextran binding include ASP115, ASN205, PHE402, ASN446, ASP447, ILE475, VAL480, and VAL481. Among them, PHE402 participates in hydrophobic interactions and ASP447 serves as the nucleophile; both are critical active-site residues at the catalytic center.

#### 3.2.4. Effect of pH on *M*_w_ Change of Dextran-H

Table S6 depicts the variation in *M*_w_ and *M*_w_/*M*_n_ over time during degradation of Dextran-H by PC-Edex under the pH range of 4.0–7.0. As shown in Table S6, the rate of change in *M*_w_ was the fastest at pH 5.0, followed by the second fastest reaction rate at pH 4.0 and 6.0, and the slowest reaction rate at pH 7.0. This may be attributed to the enzymatic properties of PC-Edex. Wang et al. [17] showed that the optimum pH for PC-Edex activity was 5.0, and the relative activity of PC-Edex was greater than 80% at pH 4.0 and 6.0; PC-Edex activity was considerably inhibited at pH 7.0. Collectively, this indicates that acidic conditions are more conducive to PC-Edex activity, whereas neutral pH conditions are less conducive to PC-Edex activity. Thus, pH 5.0 was chosen as the optimum condition for the enzymatic degradation of Dextran-H by PC-Edex.

#### 3.2.5. Variation of *M*_w_ of Dextran with Different Initial *M*_w_

Using PC-Edex as the biological enzyme material, and Dextran-T70, Dextran-T100, Dextran-H and Dextran-F as substrates, the optimized degradation conditions described earlier (PC-Edex at 8 U/mL, Dextran-H at 30 mg/mL at 50 °C and pH 5.0) were used to investigate the *M*_w_ of dextran following enzymatic degradation and the variation pattern of *M*_w_/*M*_n_.

As shown in Table S7, PC-Edex could effectively modulate the degradation of dextrans with different *M*_w_, degrading them to approximately 5 kDa within 60 min and yielding products with a narrow molecular weight distribution. These molecular weight characteristics are consistent with the general molecular weight range requirements of clinical low-molecular-weight dextran. It should be noted that the kinetic parameters obtained herein are apparent values and are only suitable for qualitative trend comparison; they should not be interpreted as rigorous intrinsic kinetic constants. These results provide a reference for the optimization of enzymatic hydrolysis process parameters for dextrans with different initial *M*_w_ values [35]. At 16 min of hydrolysis, the *M*_w_ of the four different *M*_w_ of dextran converged to 20 kDa, after which the *M*_w_ of dextran changed following a similar pattern, thus indicating that *M*_w_ changes more slowly at a limit of approximately 20 kDa, which enables exerting more control on the regulation of the *M*_w_ of dextran. Collectively, these results indicate that PC-Edex could efficiently degrade dextran at different *M*_w_ and achieve the reduction in *M*_w_ of dextran, which could be feasibly applied in the production of low-*M*_w_ dextran (<10 kDa). Moreover, the prepared low-*M*_w_ dextran shows high *M*_w_ homogeneity, reflected by *M*_w_/*M*_n_ data, which lays a foundation for subsequent process scale-up and further purification.

#### 3.2.6. Hydrolysis Kinetics of Dextran

**Figure 2:**
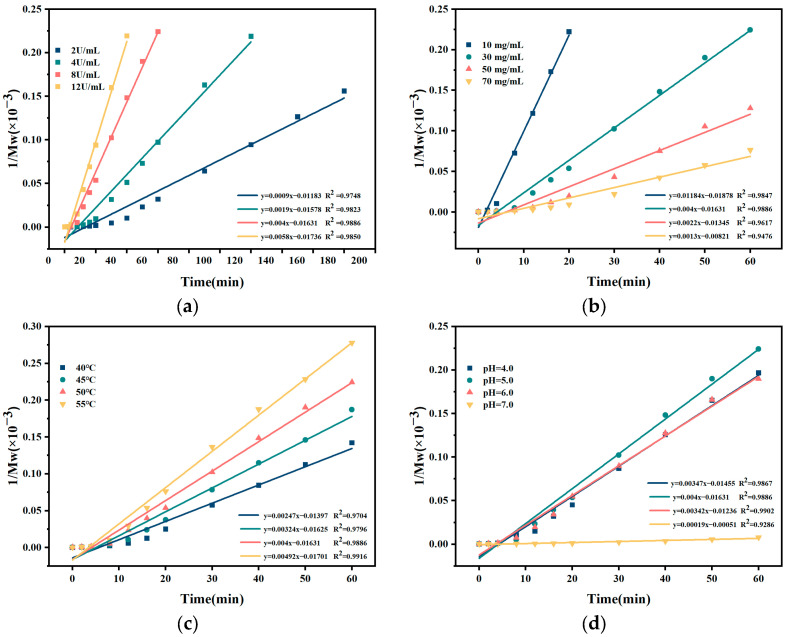
Malhotra model curve for hydrolysis of Dextran-H by PC-Edex, (**a**) concentration of PC-Edex; (**b**) concentration of dextran; (**c**) temperature; (**d**) pH.

As shown in Figure 2, the Malhotra model was applied to analyze the linear fit of 1/*M*_w_ versus time for the hydrolysis of Dextran-H by PC-Edex across all tested conditions. The full set of R^2^ values for all 16 tested conditions is provided in Table S8. Under the optimized reaction condition (8 U/mL PC-Edex, 30 mg/mL Dextran-H, 50 °C, pH 5.0), the degradation process showed high consistency with the Malhotra model, with a fitting coefficient R^2^ = 0.9886, indicating that the degradation behavior follows a second-order empirical pattern under optimal conditions. However, the goodness of fit varied with reaction parameters, with R^2^ values ranging from 0.929 to 0.992 across all tested settings. The model delivers the highest predictive reliability within the optimized process parameter range of this study.

#### 3.2.7. Change in Mass Fraction of *M*_w_ Fragments

**Figure 3:**
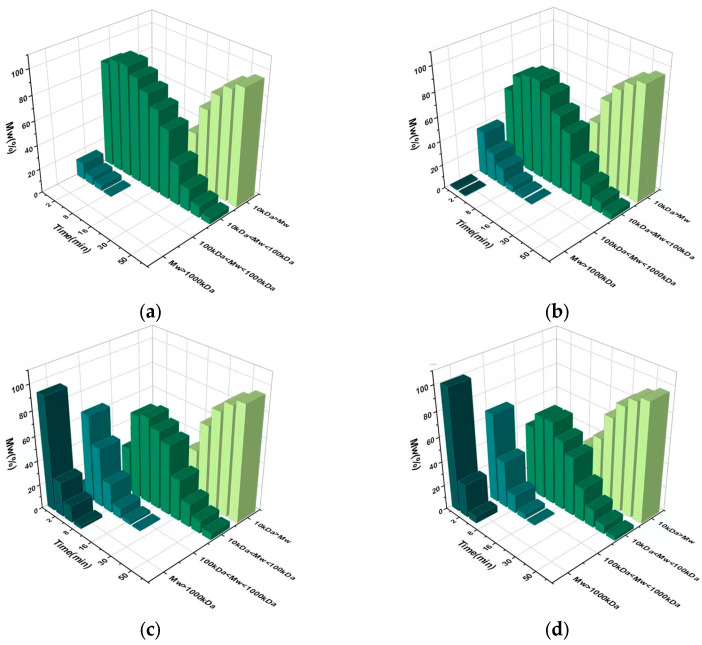
Hydrolysis of dextran fragments of different masses by PC-Edex, i.e., *M*_w_ > 1000 kDa; 100 kDa < *M*_w_ < 1000 kDa; 10 kDa < *M*_w_ < 100 kDa; and *M*_w_ < 10 kDa. (**a**) Dextran-T70; (**b**) Dextran-T100; (**c**) Dextran-H; (**d**) Dextran-F.

Enzymatic hydrolysis is the process through which high-*M*_w_ fragments of dextran are degraded to lower-*M*_w_ fragments, and the percentage of *M*_w_ fragments changes with the duration of enzymatic degradation. In the present study, the *M*_w_ fragments of dextran were classified into four categories according to their size, i.e., >1000 kDa, 100–1000 kDa, 10–100 kDa, and <10 kDa. The optimized experimental conditions were used to investigate the variations in fragment mass fraction for PC-Edex degradation of dextran at different *M*_w_, and the results are shown in Figure 3. PC-Edex degraded high-*M*_w_ Dextran-H and Dextran-F, and completely degraded all *M*_w_ fragments greater than 1000 kDa within 8 min, and fragments of 100–1000 kDa within 20 min, and fragments within 10–100 kDa were incompletely degraded between 4 and 60 min. This could be due to the fact that higher-*M*_w_ dextran chains are more prone to degradation, therefore higher-*M*_w_ fragments of dextran could be completely degraded. Since the aim of this study was to produce low-*M*_w_ dextran fragments (<10 kDa) that could be used for clinical applications after further purification, PC-Edex was used to degrade four types of dextran, and the percentage of dextran < 10 kDa was 94.56–96.80% after 60 min of reaction. The changes in the mass fraction of dextran fragments with <10 kDa showed a similar pattern in the late stages of enzymatic degradation, thus indicating that it could be considered an effective method in low-*M*_w_ dextran production.

#### 3.2.8. Comparison of Hydrolysis Rates of Dextrans with Different Molecular Weights

**Figure 4:**
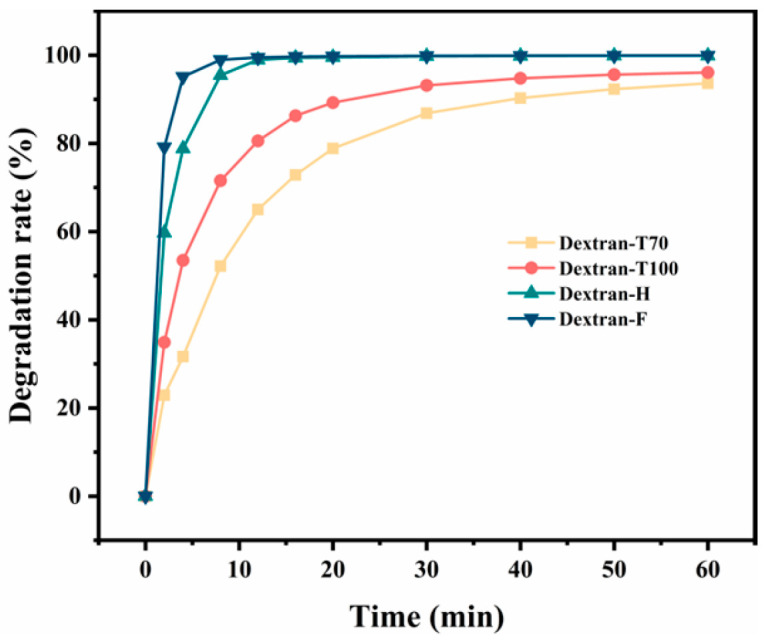
Hydrolysis rates of different molecular weight dextrans (Dextran-T70, Dextran-T100, Dextran-H, and Dextran-F) treated by PC-Edex. Reaction conditions: enzyme concentration = 8 U/mL, initial substrate concentration = 30 mg/mL, pH = 5.0, temperature = 50 °C.

In previous studies, PC-Edex exhibited the highest instantaneous catalytic efficiency at 55 °C; however, its thermal stability was poor, and it was completely inactivated after incubation at 55 °C for 1 h [17]. Taking this into comprehensive consideration, 50 °C was adopted as the enzymatic hydrolysis temperature. The degradation rates of different *M*_w_ dextran (Dextran-T70, Dextran-T100, Dextran-H and Dextran-F) treated by PC-Edex at 8 U/mL were calculated as a function of time, and the results are shown in Figure 4. The degradation rate of PC-Edex on Dextran-F (*M*_w_ = 5847.61 kDa), which has the highest *M*_w_, was the highest throughout the degradation reaction, and was the second highest on Dextran-H (*M*_w_ = 4167.16 kDa), which has a slightly lower *M*_w_. Moreover, the degradation rates of Dextran-T70, Dextran-T100, Dextran-H and Dextran-F varied widely at the early stages of degradation, and gradually slowed down as the enzymatic degradation continued. This indicates that PC-Edex has a stronger affinity for higher-*M*_w_ dextran and lower affinity for lower-*M*_w_ dextran [35]. Based on the apparent kinetic parameters obtained from the fitting, larger-molecular-weight dextran tends to present a smaller apparent *K*m value, while low-molecular-weight dextran exhibits a higher apparent *K*m value, which suggests a trend of differences in substrate affinity, although the conclusion is limited by the moderate fitting quality of the Michaelis–Menten model. Thus, PC-Edex maintained a higher degradation rate for higher-*M*_w_ dextran in the early stages of degradation, and gradually slowed down as the reaction continued, with a stabilization phase (less than 1% of increase in degradation rate) with continued enzymatic reaction. As shown in Table S9, Dextran-F, the highest-*M*_w_ dextran, reached the stabilization stage at 8 min of degradation, followed by Dextran-H, which has a slightly lower *M*_w_; Dextran-T70 and Dextran-T100, which have medium and low *M*_w_, required 40 and 50 min to reach the stabilization stage. For Dextran-H and Dextran-F, the degradation rate was greater than 98.98% and 98.96%, while for Dextran-T70 and Dextran-T100, the degradation rate was 92.32% and 94.75%, respectively. Collectively, these findings indicate that PC-Edex could degrade high-*M*_w_ dextran more completely.

### 3.3. Molecular Structure of Dextran After Enzymatic Hydrolysis

To investigate the structural changes associated with the controlled degradation process, we systematically characterized the evolution of dextran chain conformation, aggregation state and primary structure at multiple scales.

#### 3.3.1. Conformational Analysis

The *M*_w_ and [η] of a polysaccharide in solution can be expressed by the following Mark–Houwink Equation (11):

(11) $$\left[\eta \right]=KM_{v}^{\alpha }$$

In the formula, M*v* denotes the viscosity-average molecular weight, α is related to the molecular conformation of the polysaccharide, and exponential α indicates the molecular conformation of polysaccharide in aqueous solution. K is the Mark–Houwink constant that depends on the polymer–solvent system and temperature. When α decreases, it indicates that the flexibility of the polysaccharide chain is increased; when α is below 0.3, it indicates that the polysaccharide chain has a uniform spherical structure; an α value within the range of 0.5–0.8 indicates that the polysaccharide chain has a random coil-like structure; when the α value is higher, the rigidity of the polysaccharide chain also increases; and when α is greater than 1, it indicates that the polysaccharide chain has a rigid rod structure [36].

Based on Einstein’s viscosity theory and the derived hydrodynamic radius calculation framework, the following relationship between R*_h_* and *M*_w_ is established [37], as shown in Equation (12):

(12) Rh=(3Mw[η]10NAπ)13

In which *a* is the scaling exponent of the hydrodynamic radius with molecular weight (i.e., the slope of the log–log plot of R*_h_* versus *M_w_*), and when *a* is below 0.3, it suggests the polysaccharide has a uniform spherical structure; when *a* is within the range of 0.5–0.6, it indicates that the polysaccharide is a flexible irregular coil; and when *a* is greater than 1, it suggests that the polysaccharide is a rigid rod structure or a rigid chain structure.

**Figure 5:**
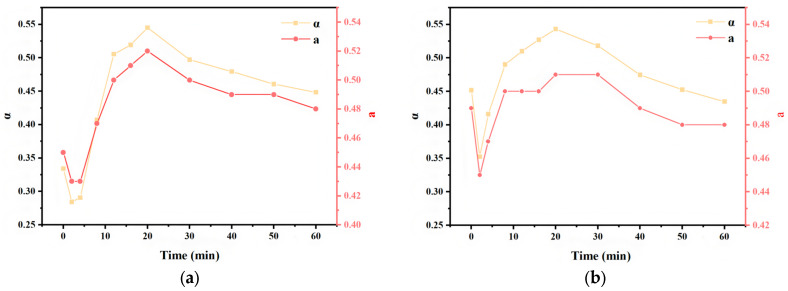
Changes in the scaling exponent a (for R*_h_* vs. *M*_w_) and the Mark–Houwink exponent α (for [*η*] vs. *M_v_*) of dextran during hydrolysis by PC-Edex: (**a**) Dextran-H and (**b**) Dextran-F degraded by PC-Edex.

As shown in Figure 5, when Dextran-H and Dextran-F were degraded by PC-Edex, *a* and α values initially decreased, then increased, and finally decreased again, thus indicating that these two types of dextran were more likely to agglomerate into a compact coil when at high *M*_w_, and the dextran chain gradually became loose upon PC-Edex degradation. After 12–30 min of degradation, when *a* and α values of dextran were approximately 0.5, dextran assumed the loosest chain structure within the *M*_w_ range of 8.29–42.91 kDa in aqueous solution. Moreover, as shown in Figure 5, both the scaling exponent *a* (for R*_h_* vs. *M*_w_) and the Mark–Houwink exponent α (for [*η*] vs. *M_v_*) values were generally in the range of 0.3–0.5, indicating that dextran existed in a compact random coil conformation in aqueous solution, which is consistent with previous reports on dextran solution properties. This compact random coil conformation provides an important structural basis for dextran to be used as a plasma substitute and iron carrier [37].

#### 3.3.2. SEM and AFM Analysis

**Figure 6:**
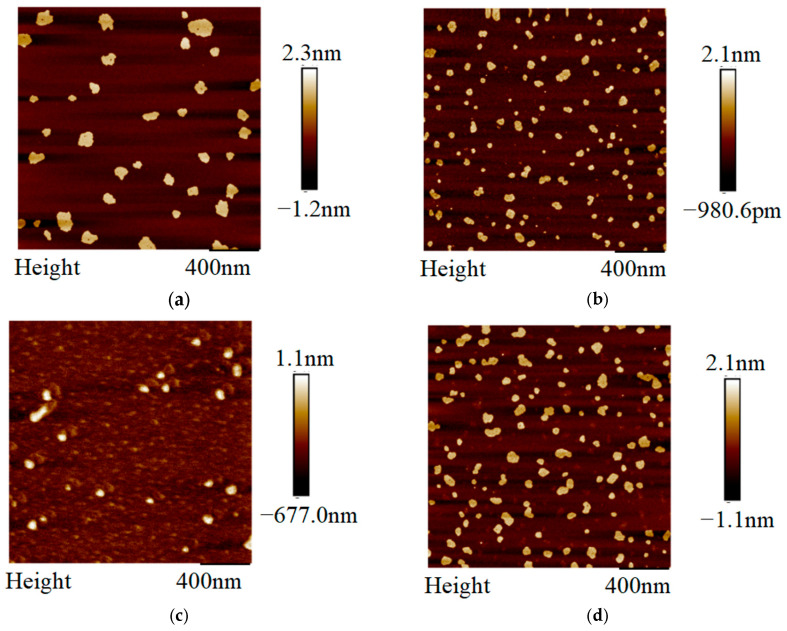
AFM images of dextran. (**a**–**d**) Planar structure of standard Dextran-H and dextran hydrolyzed by PC-Edex (*M*_w_ = 33.17 kDa, *M*_w_ = 19.97 kDa, *M*_w_ = 4.03 kDa).

**Figure 7:**
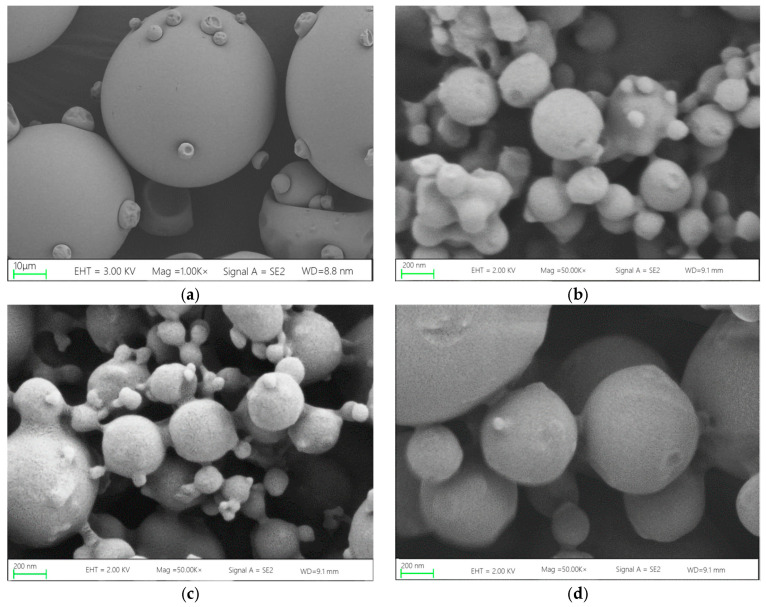
SEM images of dextran. (**a**) Standard Dextran-H at 1000× magnification. (**b**–**d**) Hydrolysis of dextran by PC-Edex (*M*_w_ = 33.17 kDa, *M*_w_ = 19.97 kDa, and *M*_w_ = 4.03 kDa) at 50,000× magnification.

AFM and SEM are commonly used to explore the surface morphology of polymeric materials, and can reveal physical and chemical properties of polysaccharides, whose microstructural properties determine their potential applications in different fields [38,39]. To investigate the morphological changes induced by enzymatic degradation, we performed AFM and SEM analyses on standard Dextran-H and enzymatically degraded dextran samples. As shown in Figure 6 and Figure 7, dextran molecules tended to aggregate into spherical-like particles in the dry state [40], which was caused by the intermolecular hydrogen bonding of polysaccharide chains during the sample drying process. This aggregation behavior indicates that dextrans of all four molecular weights have a certain dispersibility in aqueous solution [39,41]. High-*M*_w_ dextran showed a stronger aggregation tendency due to the larger number of hydroxyl groups on its longer polysaccharide chains, while low-*M*_w_ dextran after enzymatic degradation formed smaller and more uniform aggregates. Previous studies have reported that exopolysaccharides produced by *Lactobacillus kunkeei* AK1 [41] and *Streptococcus thermophilus* CC30 [42] were also regular spheres with compact morphology. Considering the morphology of polysaccharides, it could be observed that the molecules of high-*M*_w_ standard Dextran-H aggregated more strongly, whereas low-*M*_w_ dextran after enzymatic degradation aggregated less, presumably as a consequence of hydrogen bonding in polysaccharides, which could be attributed to the ability of hydroxyl groups on the polysaccharide chains to establish strong interactions between and within polysaccharide molecules [38]. Thus, combining AFM and SEM analyses, it can be concluded that dextran molecules form sphere-like aggregates in the dry state, and high-*M*_w_ dextran-H showed a larger aggregate size due to stronger intermolecular interactions, whereas low-*M*_w_ dextran after enzymatic degradation formed smaller and more uniform aggregates.

#### 3.3.3. FT-IR and NMR Analysis

FT-IR and NMR are powerful techniques for the study of functional groups and glycosidic bond types in polysaccharides. Thus, the molecular structure of enzymatically degraded dextran was analyzed by FT-IR coupled with NMR (^1^H and ^13^C).

**Figure 8:**
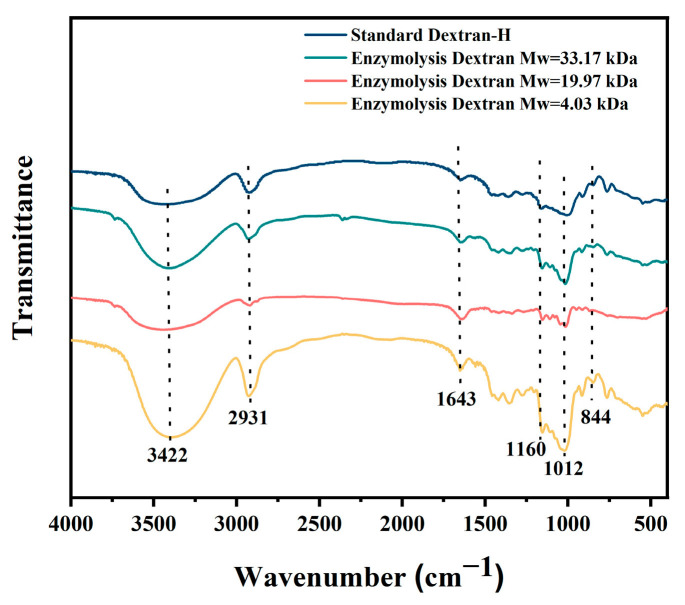
FT-IR spectra of dextran hydrolyzed by PC-Edex and standard Dextran-H.

As shown in Figure 8, two main peaks were observed at 3422 cm^−1^ and 2931 cm^−1^, which were caused by the stretching vibration of O-H and C-H [39,41]; the peak at 1643 cm^−1^ was generated by the stretching vibration of C=O and C-O [43]; the peaks at 1457 cm^−1^ and 1361 cm^−1^ were generated by the denatured absorption of C-H. Specifically, 1000–1200 cm^−1^ is the characteristic absorption peak of polysaccharide [44], whereas 1160 cm^−1^ is caused by the stretching vibration of C-O on the sugar ring; the absorption peak at 1012 cm^−1^ corresponds to α-(1 → 6) glycosidic bond [45].

**Figure 9:**
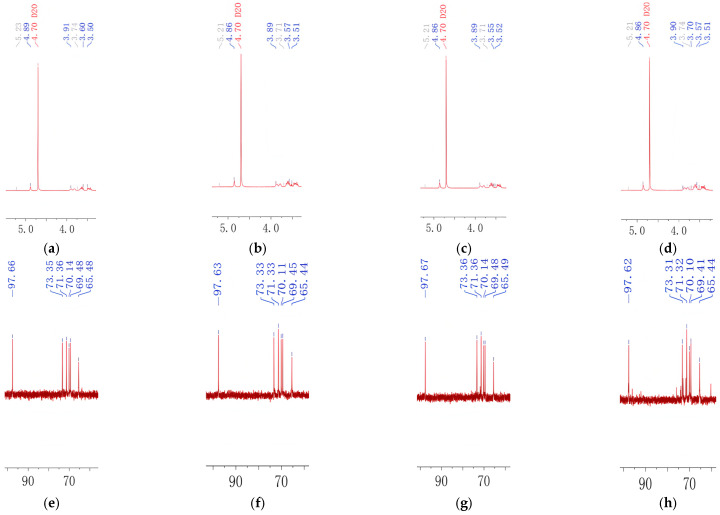
NMR spectra of dextran. (**a**–**d**) ^1^H NMR spectrum of standard Dextran-H and dextran hydrolyzed by PC-Edex (*M*_w_ = 33.17 kDa, *M*_w_ = 19.97 kDa, and *M*_w_ = 4.03 kDa); (**e**–**h**) ^13^C NMR spectrum of standard Dextran-H and dextran degraded by PC-Edex (*M*_w_ = 33.17 kDa, *M*_w_ = 19.97 kDa, and *M*_w_ = 4.03 kDa).

The ^1^H NMR spectrum of dextran consists of the heterohead region (4.5–5.5 ppm), the cyclic proton region (3.1–4.5 ppm) and the alkyl region (1.2–2.3 ppm) [46]. In the ^1^H NMR spectrum (Figure 9a–d), the peak at 4.70 ppm was assigned to deuterated water. A high-intensity signal was observed at 4.86–4.89 ppm, which corresponds to the H-1 of the α-1,6-linked D-glucose residue in the dextran backbone. In the ^13^C NMR spectrum (Figure 9e–h), the α-type glycosidic bond was mainly distributed at 97–102 ppm, the β-type glycosidic bond was mainly distributed above 102 ppm, and the heterocapital carbon was distributed at 95–110 ppm [47]. Additionally, the heterohead carbon signal was observed at 97.62–97.67 ppm as a characteristic peak for C-1 in the α-(1 → 6) glycosidic bond. Dextran usually has anomalous 13C signals at fields below 90 ppm, while C-2, C-3, C-4 and C-5 appear in the region of 70–75 ppm, and C-6 usually appears at fields near 60 ppm [46]. The peaks observed at 73.31–73.36, 71.32–71.36, 70.10–70.14 and 69.41–69.48 ppm were assigned to C-3, C-2, C-5 and C-4 substituted glucose residues, respectively. The C-6 signal of the glucose unit could be observed at 65.44–65.49 ppm instead of 60 ppm, indicating that the two glucose units in the dextran backbone were linked via an α-(1 → 6) glycoside bond [48]. FT-IR and NMR analyses confirmed that PC-Edex is an endo-dextranase that specifically cleaves α-(1 → 6) glycosidic bonds, and that the main chain structure of dextran remained unchanged after enzymatic degradation, which is consistent with previous studies on fungal dextranases. XRD and TG analyses further showed that enzymatic degradation did not alter the amorphous structure and thermal stability of dextran (Figures S2 and S3). These results are of great significance for the quality control of pharmaceutical dextran, as they ensure the structural integrity and biological safety of the product.

## 4. Discussion

In this study, an efficient enzymatic process was developed with PC-Edex from *Penicillium cyclopium* CICC-4022 to prepare controlled low-*M*_w_ dextran for potential pharmaceutical use. The optimal process parameters were determined as follows: enzyme concentration 8 U/mL, substrate concentration 30 mg/mL, reaction temperature 50 °C, and pH 5.0. Under these conditions, dextran with *M*_w_ < 10 kDa was obtained after 60 min of reaction, with the mass fraction of <10 kDa fragments exceeding 94.5% and the hydrolysis rate exceeding 98.9%.

A staged empirical regulation method based on the Malhotra model was established, to support *M*_w_ prediction and monitoring. This second-order kinetic pattern mainly arises from the chain-length dependence of PC-Edex endo-cleavage, and the system does not satisfy the premise of pseudo-first-order kinetics due to continuous chain scission. The progressive narrowing of the molecular weight distribution is mainly driven by the preferential degradation of long-chain substrates by the endo-acting PC-Edex, with a secondary contribution from the slowed reaction rate at low molecular weights. The multi-scale quality characterization results showed that the prepared low-*M*_w_ dextran had a narrow molecular weight distribution, maintained the typical compact random coil conformation of dextran, and retained good structural integrity and thermal stability.

Compared with the traditional acid degradation method, the enzymatic process developed in this study has the advantages of mild reaction conditions, environmental friendliness, and high product quality, and shows certain potential for further industrial application. Future research will focus on pilot-scale validation of the process and systematic purification of the final product to meet the requirements of large-scale industrial production.

## Data Availability

The original contributions presented in this study are included in the article/Supplementary Material. Further inquiries can be directed to the corresponding authors.

## Supplementary Materials

The following supporting information can be downloaded at: https://www.mdpi.com/article/10.3390/cimb48070749/s1, References [16,21,24,25,27] are cited in the Supplementary Materials.

## References

[1] Wang Q., Qi P.-X., Huang S.-X., Hou D.-Z., Xu X.-D., Ci L.-Y., Chen S. (2020). Quantitative analysis of straight-chain/branched-chain Ratio During Enzymatic Synthesis of Dextran Based on Periodate Oxidation. Biochem. Biophys. Res. Commun..

[2] Ren W., Cai R., Yan W., Lyu M., Fang Y., Wang S. (2018). Purification and Characterization of a Biofilm-Degradable Dextranase from a Marine Bacterium. Mar. Drugs.

[3] Köhler T., Kunchapu S., Vollrath A., Rezaei K., Kimmig J., Stumpf S., Hoeppener S., Nischang I., Jablonka K.M., Schubert U.S. (2026). Predicting acetalated dextran nanoparticle features: Controlled synthesis, formulation, and testing in a high-throughput process. Carbohydr. Polym..

[4] Lu Y., Yu Y., Pang Q., Wang X., Lu H., Zeng Z., Song Y., Feng X., Li J., Kang W. (2026). Injectable dynamic hydrogel formed with oxidized dextran and lysozyme for endometrial repair. Carbohydr. Polym..

[5] Chaudhary R., Jain S., Muralidhar K., Gupta M.N. (2006). Purification of bubaline luteinizing hormone by gel filtration chromatography in the presence of blue dextran. Process Biochem..

[6] Lin K., Du P., Dong P., Wang Y., Guo Y., Cao J., Cheng Y., Cheng F., Zhao W., Feng C. (2025). Synergistic effect of dextran and ergosterol: A venue for fabricating a water-in-oil pickering emulsion gel as a solid fat substitute in cream cheese. Food Hydrocoll..

[7] Wu Y., Sapkota A., Wilson S.N., Thompson S.G., Garren M.R., Handa H., Brisbois E.J. (2026). Nitric oxide-releasing dextran surface with enhanced albumin affinity mitigates infection and foreign body reaction. Carbohydr. Polym..

[8] Boucher A.A., Bedel A., Jones S., Lenahan S.F., Geer R., McGann P.T. (2021). A retrospective study of the safety and efficacy of low molecular weight iron dextran for children with iron deficiency anemia. Pediatr. Blood Cancer.

[9] Dai Y., Wang Z., Wang Z., Dong M., Wang D., Xia X. (2025). Stabilizing effect of Leuconostoc mesenteroides Lm10 produced dextran in situ on stirred soy yogurt: Structure-function relationship. Carbohydr. Polym..

[10] Moore R.M., Bertone A.L., Muir W.W. (1996). Effect of high-molecular weight dextran macromolecules on low-flow ischemia and reperfusion of the large colon in horses. Am. J. Vet. Res..

[11] Ioan C.E., Aberle T., Burchard W. (2000). Structure Properties of Dextran. 2. Dilute Solution. Macromolecules.

[12] Liu M., Hao Y., Wang S., Li S., Zhou J., Wang M., Zhang L., Kang X., Lyu M., Wang S. (2024). Heterologous overproduction of a dextranase in *Bacillus subtilis* WB600 and its application in preparation of porous buckwheat starch. Food Biosci..

[13] Pu Y., Zou Q., Hou D., Zhang Y., Chen S. (2017). Molecular weight kinetics and chain scission models for dextran polymers during ultrasonic degradation. Carbohydr. Polym..

[14] Zhang Y., Liu J., Hu G., Hu X., Yang J., Zhang H. (2022). Fusion enzyme design based on the “channelization” cascade theory and homogenous dextran product improvement. Int. J. Biol. Macromol..

[15] Müller O., Wefers D. (2025). Detailed insights into the oligo- and polymeric products formed by three recombinant dextransucrases. Carbohydr. Res..

[16] Huang R., Zhong L., Xie F., Wei L., Gan L., Wang X., Liao A. (2019). Purification, Characterization and Degradation Performance of a Novel Dextranase from Penicillium cyclopium CICC-4022. Int. J. Mol. Sci..

[17] Wang X., Zhang Y., Li M., Qin Q., Xie T. (2022). Purification and characterization of dextranase from Penicillium cyclopium CICC-4022 and its degradation of dextran. Int. J. Biol. Macromol..

[18] Liu H., Ren W., Ly M., Li H., Wang S. (2019). Characterization of an Alkaline GH49 Dextranase from Marine Bacterium Arthrobacter oxydans KQ11 and Its Application in the Preparation of Isomalto-Oligosaccharide. Mar. Drugs.

[19] Chen H., Pu Y., Zou Q., Hou D., Chen S. (2021). Enzymatic degradation of aqueous dextrans as affected by initial molecular weight and concentration. Polym. Bull..

[20] Eggleston G., Triplett A. (2024). Optimized Application of Dextranase at Low Doses and Retention Times to Hydrolyze Dextran in Sugarcane Juices. Sugar Tech.

[21] Yuan Y., Lan Y.Y., Huang C., Li M., Liao A.P. (2018). Optimization of dextran biosynthesis by Leuconostoc mesenteroides using response surface methodology. Food Res. Dev..

[22] Lorimer J.P., Mason T.J., Cuthbert T.C., Brookfield E.A. (1995). Effect of ultrasound on the degradation of aqueous native dextran. Ultrason. Sonochem..

[23] Wang Q., Liu T., Xu X., Chen H., Chen S. (2021). Dextran degradation by sonoenzymolysis: Degradation rate, molecular weight, mass fraction, and degradation kinetics. Int. J. Biol. Macromol..

[24] Abdelwahed N.A.M., Ahmed E.F., El-Gammal E.W., Hawas U.W. (2013). Application of statistical design for the optimization of dextranase production by a novel fungus isolated from Red Sea sponge. 3 Biotech.

[25] Miller G.L. (1959). Use of Dinitrosalicylic Acid Reagent for Determination of Reducing Sugar. Anal. Chem..

[26] Bashari M., Jin Z., Wang J., Zhan X. (2016). A novel technique to improve the biodegradation efficiency of dextranase enzyme using the synergistic effects of ultrasound combined with microwave shock. Innov. Food Sci. Emerg. Technol..

[27] Carlsson N., Borde A., Wölfel S., Åkerman B., Larsson A. (2011). Quantification of protein concentration by the Bradford method in the presence of pharmaceutical polymers. Anal. Biochem..

[28] Wang Y., Wang G., Moitessier N., Mittermaier A.K. (2020). Enzyme Kinetics by Isothermal Titration Calorimetry: Allostery, Inhibition, and Dynamics. Front. Mol. Biosci..

[29] Wu D.-T., Zhang H.-B., Huang L.-J., Hu X.-Q. (2011). Purification and characterization of extracellular dextranase from a novel producer, Hypocrea lixii F1002, and its use in oligodextran production. Process Biochem..

[30] Arnold W.N., Nguyen T.B.P., Mann L.C. (1998). Purification and characterization of a dextranase from Sporothrix schenckii. Arch. Microbiol..

[31] Mattice W.L., Riser J.M., Clark D.S. (1976). Conformational properties of the complexes formed by proteins and sodium dodecyl sulfate. Biochemistry.

[32] Chen L., Zhang B.-B., Chen J.-L., Cheung P.C.K. (2014). Cell wall structure of mushroom sclerotium (Pleurotus tuber-regium): Part 2. Fine structure of a novel alkali-soluble hyper-branched cell wall polysaccharide. Food Hydrocoll..

[33] Bhatia S., Bhakri G., Arora M., Batta S.K., Uppal S.K. (2016). Kinetic and Thermodynamic Properties of Partially Purified Dextranase from Paecilomyces lilacinus and Its Application in Dextran Removal from Cane Juice. Sugar Tech.

[34] Zhang Y., Zhang D., Li M., Qin Q., Jin Y., Fang Y., Sun G. (2023). Molecular docking and dynamics of a dextranase derived from Penicillium cyclopium CICC-4022. Int. J. Biol. Macromol..

[35] Yang L., Zhou N., Tian Y. (2018). Purification, characterization, and biocatalytic potential of a novel dextranase from Chaetomium globosum. Biotechnol. Lett..

[36] Muthukrishnan S., Mori H., Müller A.H.E. (2005). Synthesis and Characterization of Methacrylate-Type Hyperbranched Glycopolymers via Self-Condensing Atom Transfer Radical Copolymerization. Macromolecules.

[37] Harding S.E., Abdelhameed A.S., Morris G.A. (2011). On the hydrodynamic analysis of conformation in mixed biopolymer systems. Polym. Int..

[38] Kong L., Yu L., Feng T., Yin X., Liu T., Dong L. (2015). Physicochemical characterization of the polysaccharide from Bletilla striata: Effect of drying method. Carbohydr. Polym..

[39] Yang Y., Feng F., Zhou Q., Zhao F., Du R., Zhou Z., Han Y. (2018). Isolation, purification and characterization of exopolysaccharide produced by Leuconostoc pseudomesenteroides YF32 from soybean paste. Int. J. Biol. Macromol..

[40] Saravanan C., Shetty P.K.H. (2016). Isolation and characterization of exopolysaccharide from Leuconostoc lactis KC117496 isolated from idli batter. Int. J. Biol. Macromol..

[41] Yilmaz M.T., İspirli H., Taylan O., Bilgrami A.L., Dertli E. (2022). Structural and bioactive characteristics of a dextran produced by Lactobacillus kunkeei AK1. Int. J. Biol. Macromol..

[42] Kanamarlapudi S.L.R.K., Muddada S. (2017). Characterization of Exopolysaccharide Produced by Streptococcus thermophilus CC30. BioMed Res. Int..

[43] İspirli H., Sagdic O., Yılmaz M.T., Dertli E. (2019). Physicochemical characterisation of an α-glucan from Lactobacillus reuteri E81 as a potential exopolysaccharide suitable for food applications. Process Biochem..

[44] Xu X., Peng Q., Zhang Y., Tian D., Zhang P., Huang Y., Ma L., Dia V.P., Qiao Y., Shi B. (2020). Antibacterial potential of a novel Lactobacillus casei strain isolated from Chinese northeast sauerkraut and the antibiofilm activity of its exopolysaccharides. Food Funct..

[45] Das D., Goyal A. (2014). Characterization of a noncytotoxic bacteriocin from probiotic Lactobacillus plantarum DM5 with potential as a food preservative. Food Funct..

[46] Zhou Q., Feng F., Yang Y., Zhao F., Du R., Zhou Z., Han Y. (2018). Characterization of a dextran produced by Leuconostoc pseudomesenteroides XG5 from homemade wine. Int. J. Biol. Macromol..

[47] Saravanan C., Kavitake D., Kandasamy S., Devi P.B., Shetty P.H. (2019). Production, partial characterization and antioxidant properties of exopolysaccharide α-d-glucan produced by Leuconostoc lactis KC117496 isolated from an idli batter. J. Food Sci. Technol..

[48] Han J., Hang F., Guo B., Liu Z., You C., Wu Z. (2014). Dextran synthesized by Leuconostoc mesenteroides BD1710 in tomato juice supplemented with sucrose. Carbohydr. Polym..

