# Supplementary material for: Enzymatic Degradation Behavior and Molecular Weight Regulation of Dextran: Empirical Modeling and Multi-Scale Structural Characterization
Source: Curr Issues Mol Biol. 2026 Jul 22;48(7):749. doi: 10.3390/cimb48070749 (PMC13409543; doi:10.3390/cimb48070749)
Supplement: Supplementary file 1 [file cimb-48-00749-s001.zip › cimb-4403893-supplementary.pdf]

Supporting Information

Enzymatic Degradation Behavior and Molecular Weight Regulation of Dextran: Empirical Modeling and Multi-Scale Structural Characterization

Mei Li [†],Piaoran Fan [†],Yirui Zhang , Ranran Li ,Lemin Chen , Donghui Zhang, Lei Zhong *

Guangxi Key Laboratory of Polysaccharide Materials and Modification, School of Chemistry and Chemical Engineering, Guangxi Minzu University, Nanning 530006, China; meili@gxmzu.edu.cn (M.L.); 15516442022@163.com (P.F.); 2023200216@snnu.edu.cn (Y.Z.); lr051200@163.com (R.L.); cleminn@163.com (L.C.); 15232157763@163.com (D.Z.)

* Correspondence: 20040055@gxmzu.edu.cn

[†] These authors contributed equally to this work.

Supplementary Note S1. Production and purification of PC-Edex from *Penicillium cyclopium* CICC-4022

The following mixture was used as the fermentation medium for *P. cyclopium* CICC-4022: dextran T20, 30 g/L; yeast extract, 4 g/L; KCl, 0.5 g/L; $MgSO_4$, 0.5 g/L; $FeSO_4$, 0.1 g/L; and K_2HPO_4, 1 g/L; pH, 6.0 [1]. Fermentation was conducted under the following parameters: bioreactor total volume, 2.5 L; inoculated volume, 1 L; duration of fermentation, 72 h; temperature of fermentation, 30 °C; gas volume, 2 vvm; inoculation ratio, 6.5%; rotation speed, 350 rpm; and defoamer, 1 mL. Crude PC-Edex from *P. cyclopium* CICC-4022 was extracted by centrifugation at 4 ℃ and 10,000 rpm for 15 min, and the solution was subsequently stored at 4 ℃.

A unit of PC-Edex activity (1U) was determined based on the amount of PC-Edex to degrade dextran T70 and produce 1 mg of reducing sugar within 1 h [2, 3]. PC-Edex activity was calculated based on equation (S1) as follows:

$$\text{PC} - \text{Edex activity}(\text{U/m L}) = \frac{\text{Amount of reducing sugar (mg)} \times \text{Dilution factor of PC} - \text{Edex solution}}{\text{Volume of PC} - \text{Edex (mL)} \times \text{Time (h)}} \quad \text{(S1)}$$

Protein concentration (mg/mL) was determined utilizing the Bradford method and crystallized bovine serum albumin as the protein standard [4].

Briefly, 80% ammonium sulfate salting-out process was used to obtain crude PC-Edex solution, and 20 mM acetic acid buffer solution (pH 5.0) was used to dissolve PC-Edex in the precipitate. Purification of PC-Edex by tangential flow filtration. A Pall Minimate™ TFF system (Pall Corporation, Port Washington, NY, USA) and a polyethersulfone membrane package (Pall Corporation) with a 50-cm^2 surface area were used for ultrafiltration of PC-Edex solution, and the retentate was collected with 10–100 K Minimate™ TFF capsules.

The purity and molecular mass of PC-Edex were determined by SDS-PAGE. A protein marker was used as the control sample, and 15 μL of samples were loaded onto 12% of precast SDS-PAGE gel. Samples were dyed with fast blue protein gel staining solution for 1 h. PC-Edex was recovered by gel cutting and further purified using a PAGE gel protein micro-recovery kit, and its molecular weight was determined using MATLAB-TOF.

Supplementary Note S2. Production and purification of Dextran-F from *Leuconostoc mesenteroides*

The fermentation medium optimized by Yuan et al[5] was used for the production of dextran: sucrose, 70 g/L;

peptone, 7 g/L; disodium hydrogen phosphate, 1.4 g/L; potassium dihydrogen phosphate, 0.3 g/L; pH, 7.0. Fermentation was conducted under the following parameters: bioreactor total volume, 2.5 L; inoculated volume, 1 L; duration of fermentation, 24 h; temperature of fermentation, 30 °C; gas volume, 2 vvm; inoculation ratio, 10%; rotation speed, 200 rpm. The fermentation broth was diluted by three times the mass volume ratio, and centrifuged at 4 ℃ for 20 min in a high-speed centrifuge at 11000 rpm to remove bacteria; the protein in the fermentation broth was precipitated using trifluoroacetic acid (TCA) with a mass fraction of 20%, and centrifuged at 4 ℃ for 20 min in a high-speed centrifuge at 11000 rpm. The supernatant was transferred to a dialysis bag with a molecular weight cutoff of 13 kDa to remove small molecule impurities, and dialysis occurred for 48 h. Distilled water was replaced once at 4 h in the early stage of dialysis, and once at 12 h in the late stage of dialysis. The Dextran-F solution purified by dialysis was mixed with anhydrous ethanol at a ratio of 1:3 (*v*/*v*), and left for 24 h to separate and allow for dextran precipitation. The Dextran-F was dried in an oven at 55 ℃.

Supplementary Note S3. X-ray diffractometry (XRD)

A MiniFlex600 X-ray diffractometer (Rigaku Corporation, Tokyo, Japan) was used to analyze dextran of varying molecular weights under the following conditions: scanning rate, 8; step width, 0.02; voltage, 40 Kv; current, 15 mA; scanning range, 2θ=3-80°.

Supplementary Note S4. Thermogravimetric (TG) analysis

Thermal decomposition and combustion performance of dextran products were analyzed in a TGA5500 synchronous thermal analyzer (TA Instruments, New Castle, DE, USA). Operating conditions were as follows: dextran, 3-5 mg; nitrogen flow rate, 100 mL/min; heating rate, 10 °C/min; heating range, 25-800 ℃.

Supplementary Figures

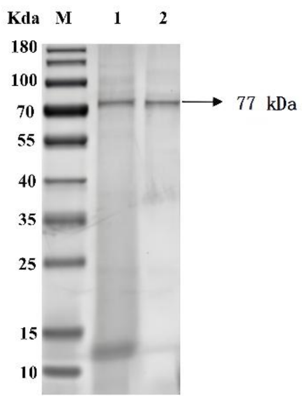

Figure S1. SDS-PAGE of PC-Edex, M: protein marker; 1: crude PC-Edex solution; 2: PC-Edex purified by tangential flow filtration. Faint miscellaneous bands in Lane 1 were minor secreted proteins removed during purification; the single-band purified enzyme (Lane 2) was used for all formal experiments.

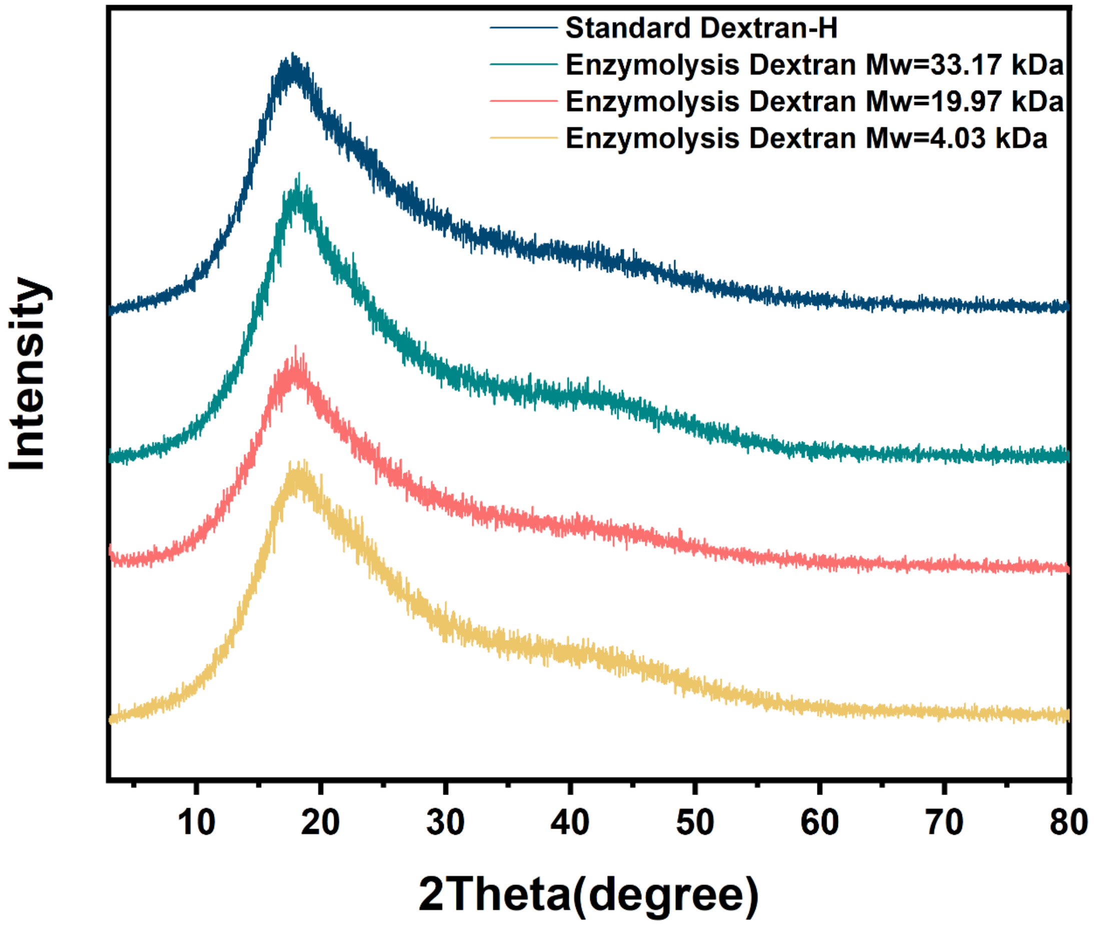

Figure S2. XRD spectra of dextran degraded by PC-Edex and standard Dextran-H.

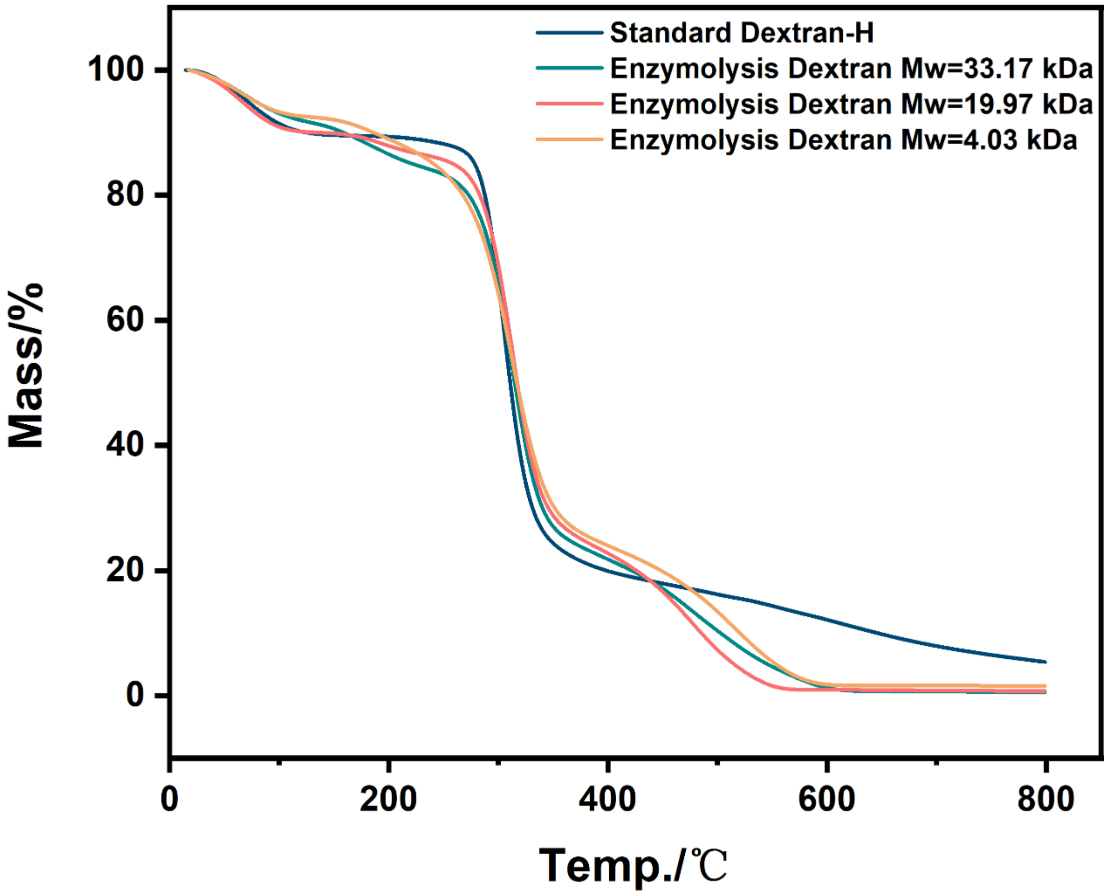

Figure S3. TG of dextran degraded by PC-Edex and standard Dextran-H.

Supplementary Tables

Table S1. Purification of PC-Edex.

Purification step	Total protein(mg)	Total activity(U)	Specific activity(U/mg)	Purification(fold)	Yield(%)
Culture PC-Edex	5.00	15606.64	3121.33	1.00	100.00
$(NH_4)_2SO_4$ Precipitation	3.77	13615.84	3611.63	1.16	87.24
Tangential flow filtration	0.85	9757.71	11479.66	3.68	62.52

Table S2. Mw and Mw/Mn of Dextran-H under different PC-Edex (dextranase) activity.

Reaction time (min)	2 U/mL		4 U/mL		8 U/mL		12 U/mL	
	Mw(kDa)	Mw/Mn	Mw(kDa)	Mw/Mn	Mw(kDa)	Mw/Mn	Mw(kDa)	Mw/Mn
0	4167.16	2.078	4167.16	2.078	4167.16	2.078	4167.16	2.078
2	3405.92	2.279	2660.2	2.544	1678.3	4.169	1180.31	6.076
4	2920.24	2.49	1873.16	3.436	882.82	8.332	351.27	10.056
8	1921.23	3.395	845.88	6.619	189.43	5.998	66.58	5.115
12	1247.18	5.938	378.06	8.846	42.91	2.799	23.22	2.677
16	823.13	8.793	181.36	7.873	25.25	2.627	14.48	2.694
20	534.21	8.194	104.51	4.725	18.64	1.998	10.65	2.024
30	212.05	5.894	31.53	2.855	9.78	1.757	6.25	1.622
40	96.68	4.537	19.55	2.469	6.74	1.708	4.56	1.556
50	43.09	2.523	13.69	2.078	5.26	1.619	3.83	1.408
60	31.32	2.265	10.28	2.01	4.46	1.528		
90	15.57	2.604	6.14	1.868				
120	10.59	1.825	4.57	1.578				
150	7.91	1.812						
180	6.41	1.604						

Table S3. Mw and Mw/Mn of Dextran-H under different substrate concentrations.

Reaction time (min)	10 mg/mL		30 mg/mL		50 mg/mL		70 mg/mL	
	Mw(kDa)	Mw/Mn	Mw(kDa)	Mw/Mn	Mw(kDa)	Mw/Mn	Mw(kDa)	Mw/Mn
0	4167.16	2.078	4167.16	2.078	4167.16	2.078	4167.16	2.078
2	455.9	9.108	1678.3	4.169	2288.97	2.965	2280.91	3.244
4	94.66	3.511	882.82	8.332	1350.54	5.201	1508.66	4.624
8	13.77	2.479	189.43	5.998	488.28	8.772	674.99	8.243
12	8.23	1.787	42.91	2.799	180.52	6.034	316.52	9.141
16	5.78	1.556	25.25	2.627	83.53	4.593	176.43	5.971
20	4.5	1.547	18.64	1.998	50.93	3.613	107.77	4.92
30	2.82	1.276	9.78	1.757	23.19	2.502	45.19	3.032
40			6.74	1.708	13.3	2.394	23.58	2.528
50			5.26	1.619	9.49	2.161	17.34	2.372
60			4.46	1.528	7.82	1.944	13.1	2.268

Table S4. Mw and Mw/Mn of Dextran-H under different temperatures.

Reaction time (min)	40℃		45℃		50℃		55℃	
	Mw(kDa)	Mw/Mn	Mw(kDa)	Mw/Mn	Mw(kDa)	Mw/Mn	Mw(kDa)	Mw/Mn
0	4167.16	2.078	4167.16	2.078	4167.16	2.078	4167.16	2.078
2	1765.52	4.093	1726.5	4.04	1678.3	4.169	1309.75	4.106
4	1138.49	6.787	892.81	6.346	882.82	8.332	543.92	8.052
8	422.95	9.062	256.85	7.179	189.43	5.998	99.21	3.97
12	164.23	6.24	91.23	4.459	42.91	2.799	33.76	2.85
16	78.63	4.039	41.86	3.457	25.25	2.627	18.6	2.242
20	39.85	2.281	26.54	2.318	18.64	1.998	13.11	1.923
30	17.41	2.335	12.73	2.002	9.78	1.757	7.34	1.793
40	11.83	2.088	8.71	1.764	6.74	1.708	5.33	1.69
50	8.88	1.894	6.85	1.628	5.26	1.619	4.38	1.531
60	7.03	1.862	5.35	1.614	4.46	1.528	3.60	1.501

Table S5. Thermodynamic parameters of the interaction between PC-Edex and dextran-T70 at different temperatures obtained by ITC.

Temperature (℃)	Kd (M)	ΔHhydr (kJ/mol)
40	1.703×10^{-6}	81.29
45	1.233×10^{-6}	96.34
50	3.200×10^{-7}	99.93
55	2.473×10^{-8}	99.96

Table S6. Mw and Mw/Mn of Dextran-H under different pH values.

Reaction time (min)	pH 4.0		pH 5.0		pH 6.0		pH 7.0	
	Mw(kDa)	Mw/Mn	Mw(kDa)	Mw/Mn	Mw(kDa)	Mw/Mn	Mw(kDa)	Mw/Mn
0	4167.16	2.078	4167.16	2.078	4167.16	2.078	4167.16	2.078
2	1489.85	3.819	1678.3	4.169	1529.22	4.529	3302.98	2.429
4	790.51	7.532	882.82	8.332	688.95	7.507	2729.19	2.95
8	92.8	4.973	189.43	5.998	136.89	4.273	2122.93	3.719
12	66.87	4.162	42.91	2.799	51.16	3.626	1543.08	5.122
16	31.09	2.587	25.25	2.627	29.44	2.456	1195.33	5.98
20	22.12	2.436	18.64	1.998	18.26	2.372	863.5	7.483
30	11.51	1.856	9.78	1.757	11.15	1.825	452.98	7.43
40	7.95	1.836	6.74	1.708	7.85	1.736	282.92	5.63
50	6.05	1.658	5.26	1.619	6.03	1.680	177.58	5.406
60	5.08	1.544	4.46	1.528	5.27	1.557	128.4	4.329

Table S7. Mw and Mw/Mn of dextran based on different Mw of substrates.

Reaction time (min)	Dextran-T70		Dextran-T100		Dextran-H		Dextran-F	
	Mw(kDa)	Mw/Mn	Mw(kDa)	Mw/Mn	Mw(kDa)	Mw/Mn	Mw(kDa)	Mw/Mn
0	69.23	1.240	138.55	2.144	4167.16	2.078	5847.61	1.327
2	53.37	1.422	89.85	2.085	1678.3	4.169	1217.59	4.34
4	47.31	1.501	63.95	2.052	882.82	8.332	284.09	4.661
8	33.10	1.692	38.7	2.026	189.43	5.998	60.45	3.325
12	24.23	1.678	26.13	1.929	42.91	2.799	30.21	2.317
16	18.80	1.605	18.15	1.925	25.25	2.627	18.13	2.203
20	14.63	1.596	14.02	1.829	18.64	1.998	13.92	1.778
30	9.12	1.590	8.56	1.828	9.78	1.757	8.29	1.769
40	6.73	1.550	6.33	1.737	6.74	1.708	6.11	1.588
50	5.32	1.470	5.13	1.612	5.26	1.619	4.7	1.530
60	4.40	1.467	4.45	1.428	4.46	1.528	4.29	1.480

Table S8. Goodness of fit (R^2) of the Malhotra model for Dextran-H degradation by PC-Edex under all tested reaction conditions.

Parameter group	Reaction condition	Fitting equation ($1/Mw = kt + b$)	R^2
	2 U/mL	y=0.0009x-0.01183	0.9748
**Enzyme concentration**	4 U/mL	y=0.0019x-0.01578	0.9823
	8 U/mL (Optimal)	y=0.0040x-0.01631	0.9886

	12 U/mL	y=0.0058x-0.01736	0.9850
**Substrate concentration**	10 mg/mL	y=0.01184x-0.01878	0.9847
	30 mg/mL (Optimal)	y=0.0040x-0.01631	0.9886
	50 mg/mL	y=0.0022x-0.01345	0.9617
	70 mg/mL	y=0.0013x-0.00821	0.9476
**Temperature**	40 °C	y=0.00247x-0.01397	0.9704
	45 °C	y=0.00324x-0.01625	0.9796
	50 °C (Optimal)	y=0.00400x-0.01631	0.9886
	55 °C	y=0.00492x-0.01701	0.9916
**pH**	4.0	y=0.00347x-0.01455	0.9867
	5.0 (Optimal)	y=0.00400x-0.01631	0.9886
	6.0	y=0.00342x-0.01236	0.9902
	7.0	y=0.00019x-0.00051	0.9286

Note: 1/Mw has the unit of $\times 10^{-3}$ Da^{-1}; x represents reaction time in minutes. Conditions marked with (Optimal) correspond to the final optimized reaction parameters. $R^2 > 0.98$ is defined as the threshold for high goodness of fit in this study.

Table S9. Comparison of the minimum time required for different molecular weight substrates to reach a stable degradation rate during hydrolysis by PC-Edex at 8 U/mL.

Substrate	Stable degradation rate (%)	Minimum time required (min)
Dextran-T70	92.32	50
Dextran-T100	94.75	40
Dextran-H	98.98	12
Dextran-F	98.96	8

References

1. Huang, R.; Zhong, L.; Xie, F.; Wei, L.; Gan, L.; Wang, X.; Liao, A., Purification, Characterization and Degradation Performance of a Novel Dextranase from *Penicillium cyclopium* CICC-4022. *International Journal of Molecular Sciences* **2019,** 20, (6), 1360.
2. Abdelwahed, N. A. M.; Ahmed, E. F.; El-Gammal, E. W.; Hawas, U. W., Application of statistical design for the optimization of dextranase production by a novel fungus isolated from Red Sea sponge. *3 Biotech* **2014,** 4, (5), 533–544.
3. Miller, G. L., Use of Dinitrosalicylic Acid Reagent for Determination of Reducing Sugar. *Analytical Chemistry* **1959,** 31, (3), 426–428.
4. Carlsson, N.; Borde, A.; Wölfel, S.; Åkerman, B.; Larsson, A., Quantification of protein concentration by the Bradford method in the presence of pharmaceutical polymers. *Analytical Biochemistry* **2011,** 411, (1), 116–121.
5. Yuan, Y.; Lan, Y. Y.; Huang, C.; Li, M.; Liao, A. P., Optimization of dextran biosynthesis by Leuconostoc mesenteroides using response surface methodology. *Food Res. Dev.* **2018,** 39, (7), 187–192.
